# Is reading fiction associated with a higher mind-reading ability? Two conceptual replication studies in Japan

**DOI:** 10.1371/journal.pone.0287542

**Published:** 2023-06-22

**Authors:** Yuka Takahashi, Toshiyuki Himichi, Ayumi Masuchi, Daisuke Nakanishi, Yohsuke Ohtsubo

**Affiliations:** 1 Graduate School of Humanities, Kobe University, Kobe, Hyogo, Japan; 2 Research Center for Future Design, Kochi University of Technology, Kochi, Japan; 3 Faculty of Business Administration, Hokkai-Gakuen University, Sapporo, Hokkaido, Japan; 4 Faculty of Health Sciences, Hiroshima Shudo University, Hiroshima, Japan; 5 Graduate School of Humanities and Sociology, University of Tokyo, Tokyo, Japan; Edge Hill University, UNITED KINGDOM

## Abstract

Previous studies have revealed that reading fiction is associated with dispositional empathy and theory-of-mind abilities. Earlier studies established a correlation between fiction reading habits and the two measures of social cognition: trait fantasy (i.e., the tendency to transpose oneself into fictitious characters) and performance on the Reading the Mind in the Eyes Test (RMET; a test of the ability to identify others’ mental states based on their eyes). Recently, experimental studies have shown that brief exposure to fiction enhances RMET performance. Nevertheless, these studies have been conducted only in Western countries, and few published studies have investigated these relationships in Asian countries. This research aims to address this gap. Study 1, which involved 338 Japanese undergraduates, conceptually replicated the previously reported correlations between fiction reading and fantasy and RMET scores (after statistically controlling for the effect of outliers). However, Study 2, which involved 304 Japanese undergraduates, failed to replicate the causal relationship. Participants read an excerpt either from literary fiction or from nonfiction, or engaged in a calculation task, before completing the RMET. Brief exposure to literary fiction did not increase the RMET score. In sum, this study replicated the associations of fiction reading with fantasy and RMET scores in Japan, but failed to replicate the causal relationship.

## Introduction

Empathy has attracted the interest of researchers in diverse fields, such as philosophy [1], economics [2], neuroscience [3, 4], and animal behavior [5, 6], to mention but a few. Social psychology is no exception [7]. In social psychology, empathy has come to be regarded as a prosocial emotion that promotes altruistic behavior [8, 9] and forgiveness [10]. Researchers also regard empathy and its related concepts, such as theory-of-mind and mentalizing, as being key to understanding psychological/personality disorders, such as autism spectrum disorder (see [11] for the relation of this disorder to theory-of-mind or cognitive empathy) and psychopathy (see [12] for the relation of this disorder to affective empathy). Accordingly, many researchers are interested in factors that promote empathy.

Previous studies have revealed that reading fiction is positively associated with empathy and theory-of-mind (see [13] for a recent review), and some authors consider reading fiction to be an effective means of fostering empathy and prosocial behavior [14]. Mar et al.’s [15] seminal study on this relationship assessed participants’ reading habits, dispositional empathy, and theory-of-mind abilities. Reading habits were assessed using the Author Recognition Test (ART) [16], which requires participants to check which authors’ names they recognize on a list of author and non-author names. Dispositional empathy was assessed using a self-report measure, the Interpersonal Reactivity Index (IRI) [17]. Theory-of-mind abilities were assessed using two tasks, one of which was the Reading the Mind in the Eyes Test (RMET) [18]. The RMET, which consists of a series of photographs depicting the eye area of a person, requires participants to identify the mental state of the target person. Mar et al. [15] revealed that the reading of fiction, but not of nonfiction, was positively associated with one of the four subcomponents of IRI (i.e., fantasy) and the RMET score, which means that individuals who habitually read fiction are more likely to transpose themselves into the feelings and actions of fictitious characters (IRI fantasy) and are better at inferring others’ mental states from their eyes (RMET).

The positive correlation between reading fiction and empathy-related social cognitive processes was confirmed in subsequent studies [19–23]. A meta-analytic review by Mumper and Gerrig [24] concluded that “frequent leisure reading of fiction or nonfiction was associated with slightly higher empathy and theory-of-mind abilities” (p. 116). However, the studies included in Mumper and Gerrig’s meta-analysis [24] were conducted in the U.S., Canada, and some European countries (see Tables 1 and 2 in [24]; but see [22] for a Mexican study published after the meta-analytic review [24]), and few studies have examined this fiction–empathy association in East Asian countries. Therefore, the aim of Study 1 was to examine whether these associations would be observed in Japan, an East Asian country.

Although the correlational nature of the abovementioned studies [15, 19–24] does not allow researchers to draw any causal conclusions regarding whether habitual fiction reading improves one’s theory-of-mind ability, a recently proposed Social Processes and Content Entrained by Narrative (SPaCEN) framework [25] identifies two potential causal pathways from fiction reading to social cognitive abilities. First, narratives evoke social cognitive processing; thus frequent exposure to narratives in fiction enhances social cognition via practice effects. Second, narratives (even fiction) may contain accurate social content and allow readers to learn this content. Once learned, accurate knowledge of social content can be applied in the real world.

Although the validity of the SPaCEN framework (especially the validity of the hypothesized causal pathways) is yet to be examined, a series of experiments conducted by Kidd and Castano [26] suggested that there was a more short-term causal effect of fiction reading on theory-of-mind ability: Brief exposure to excerpts from literary fiction, as compared to popular fiction and nonfiction, improved the RMET score. On the one hand, there are successful replication studies of [26], including both exact and conceptual replications (see [27–30] for examples). On the other, there are studies that have reported replication failures (see [21, 31–34] for examples; see also [35] for mixed results). Despite such mixed evidence, based on a meta-analysis of similar experimental studies, Dodell-Feder and Tamir [36] concluded that “fiction reading leads to a small … but statistically significant improvement in social-cognitive performance compared to nonfiction reading or no reading” (p. 1724). This conclusion was recently enhanced by a follow-up *p*-curve analysis, which indicated that the statistically significant associations of fiction reading with empathy and theory-of-mind abilities are not attributable to the questionable research practice of *p*-hacking [37].

However, few studies have attempted to replicate this causal relationship in Asian countries. The goal of Study 2 of the present study was to examine whether the effect of brief exposure to literary fiction on theory-of-mind abilities (i.e., the RMET score) could also be observed in a Japanese undergraduate sample.

## Study 1

### Method

#### Participants

Participants in Study 1 were 338 undergraduate students (187 women, 143 men, and 8 unreported), and their average age (*SD*) was 19.01 (1.05) years. The data were collected at three Japanese universities (one large national university and two medium-sized private universities) in 2016. Because the meta-analysis [24] was not available when we conceived this study, we used the correlation coefficient reported in Mar et al.’s original study [15] to determine the sample size. Using the “pwr” package in R [38] (expected correlation = .20, significance level = .05 [two-sided], power = .80), we determined that the necessary sample size was 193. However, since this employed a different measure of fiction reading than Mar et al.’s study [15], we decided to recruit at least 300 participants (i.e., approximately 100 more participants than the required sample size).

#### Materials and procedure

Participants in Study 1 completed the Japanese version of the Autism-Spectrum Quotient (AQ) [39], the Japanese version of the IRI [40], the Japanese version of the RMET [41], and a questionnaire designed to assess their exposure to fiction. The AQ was originally developed to assess the degree to which an individual has traits typically associated with the autistic spectrum [42]. It was included in this study for exploratory purposes because the AQ score is known to correlate with the RMET score [18] (but see the Supplementary Material of [43] for a nonsignificant correlation between the AQ total score and the REMT score). However, we do not report the analyses including the AQ score in the main text because there was no a priori hypothesis. The results of the analyses involving AQ score are summarized in the S1 File.

The IRI was developed by Davis [17] to measure four subdimensions of dispositional empathy: empathic concern (EC: tendency to experience sympathetic feelings for someone in need), perspective taking (PT: tendency to spontaneously take another person’s point of view), personal distress (PD: tendency to feel discomfort in the presence of someone in need), and fantasy (FS: tendency to transpose oneself into the feelings and actions of fictitious characters). The IRI consists of 28 items (seven items for each of the four subdimensions), such as “I really get involved with the feelings of the characters in a novel” (FS). Participants rated how well each statement described themselves on a 5-point scale (1 = “not at all” to 5 = “very much”).

The RMET consisted of 36 photographs of the eye regions of the target persons. Each photograph was accompanied by four possible mental state terms (e.g., comforting, irritated). Participants’ task was to choose the most appropriate term to describe the mental state of each of the 36 targets. The RMET score was operationalized as the number of photographs for which the target person’s mental state was correctly identified, thus theoretically ranging from 0 to 36.

We developed a novel questionnaire to assess participants’ exposure to fiction because there was no readily available ART for Japanese samples (although a Japanese version has been developed, it was only presented at a conference [44] and has not been published). Instead of developing a Japanese version of ART, we asked participants to report the amount of time they spent reading fiction (according to [45], the correlation between the ART score and self-reported reading time was approximately .40). To obscure the purpose of the study, we included some filler questions in the fiction-reading questionnaire. In the first section, participants reported the number of hours they usually spent on sports, reading books (except *manga*), playing video games, reading *manga*, and social networking services (e.g., Facebook) in a month (except for social networking services, for which participants reported hours per day). Participants also reported the number of films that they watched (per year). For the item of primary interest (i.e., reading books), participants further reported the proportions of fiction and nonfiction in books that they usually read. For example, if a participant reported that they spent 10 hours reading books, and 60 percent of the books they read were fiction, we defined their fiction-reading time per month as 6 hours. This first section was followed by three subsidiary sections. In the second section, participants reported how much they liked each activity. In the third section, they reported details of their reading habits (e.g., genre of fiction they like). In the fourth section, they reported how many hours they spent on each activity in a month when they were first to third graders, fourth to sixth graders, junior high school students, and high school students. Although we primarily focus on the first section in the main text, the questionnaire and data of the remaining sections are available in the OSF (see Data Availability).

This study was conducted in a small-group session, and was followed by unrelated studies. Participants were paid 700–1000 Japanese yen for their participation, depending on the load of unrelated studies that were combined with this study.

### Ethics statement

This study was approved by the Ethics Committee on Research Involving Human Subjects of the Graduate School of Humanities, Kobe University, Japan. Written informed consent was obtained from all participants.

## Results and discussion

Descriptive statistics for the variables of interest are summarized in Table 1. It is obvious that fiction reading time and nonfiction reading time were highly positively skewed. Accordingly, these two variables were log transformed (after adding a small constant of 0.1) for subsequent analyses. We chose a base of 10 for the log-transformation because of its ease of interpretation: 0.1 (≈ 0), 1, 10, and 100 hours were transformed to −1, 0, 1, and 2, respectively. After this transformation, the skewness scores of fiction and nonfiction reading time were substantially reduced to −0.38 and 0.81, respectively.

**Table 1:** Descriptive statistics of the variables of interest (Study 1).

	*M*	*SD*	Min.	Max.	Skewness
Fiction Reading Time	6.66	11.50	0	100	4.33
Nonfiction Reading Time	1.40	4.00	0	49.5	7.38
Sports Time	14.33	18.07	0	92	1.91
Videogame Time^a^	39.02	55.3	0	360	2.22
*Manga* Reading Time	8.81	19.62	0	240	6.81
#Films per Year	10.27	14.66	0	100	3.61
SNS Time (per Day)^b^	2.34	2.31	0	16	2.83
RMET^c^	25.49^d^	3.42	12	33	−0.52
Perspective Taking	3.06	0.64	1.00	5.00	−0.04
Personal Distress	3.26	0.68	1.29	5.00	−0.19
Empathic Concern	3.46	0.68	1.29	4.86	−0.56
Fantasy	3.57	0.81	1.00	5.00	−0.48

^a^Two implausible datapoints (longer than 500 hours per month) were removed.

^b^SNS = social networking services. Two implausible datapoints (longer than 24 hours per day) were removed.

^c^RMET = “Reading the Mind in the Eyes Test” score.

^d^For an unknown reason, the mean RMET score (the .71 correct response rate) was slightly lower than the norm for Japanese RMET score. The Japanese RMET was developed such that the non-clinical sample exceeded the .73 correct response rate [41]. A one-sample *t*-test against the criterion of .73 indicated the difference was significant, *t*(337) = 4.25, *p* < .001.

**Fig 1:**
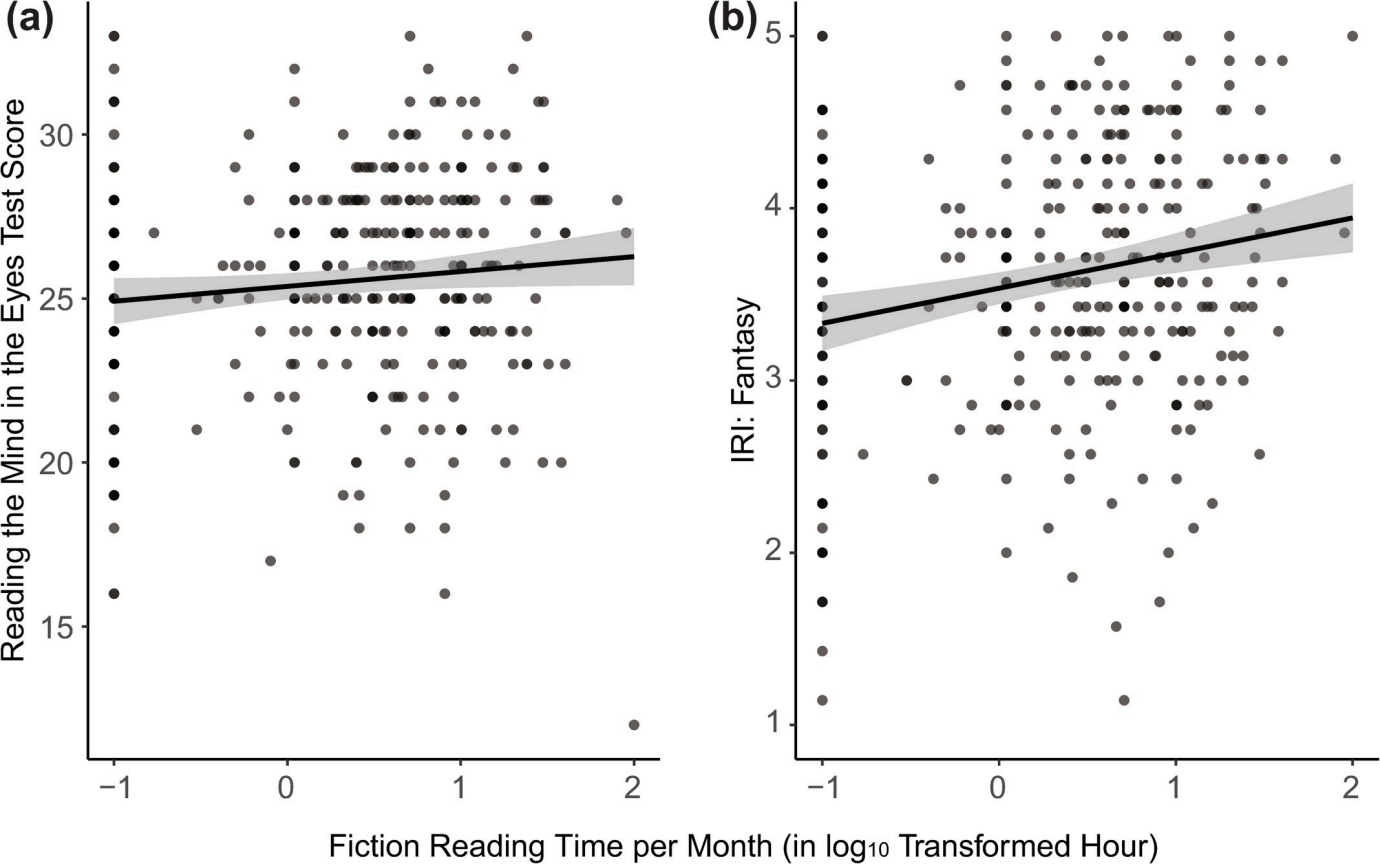
Scatter Plots Showing the Relationships between Fiction Reading Time and (a) RMET Score and (b) Fantasy Score.

The bivariate correlation coefficients between fiction and nonfiction reading times and IRI/RMET scores are summarized in Table 2. Confirming the previous results [15], fiction reading time was significantly correlated with FS: *r*(315) = .21, *p* < .001 (Fig 1B). However, the correlation between fiction reading time and RMET score failed to reach the conventional significance level: *r*(315) = .11, *p* = .052 (Fig 1A). Visual inspection of Fig 1A suggests that the failure to reach the significance level may be due to the presence of an outlier around the bottom-right corner of the plot (the outlier’s RMET score was 3.94*SD* below the mean RMET score). Instead of removing this outlier data point, we conducted a robust regression analysis [46], which assumed the *t* distribution instead of the normal distribution for the RMET score. For this analysis, we used the “rstan” package of R [47]. The standardized coefficient from the robust regression analysis was .13, with a Bayesian 95% credible interval of [.01, .23], which was associated with the sign of successful convergence of R-hat = 1 (four chains). As the 95% credible interval did not include 0, we can conclude that RMET was significantly correlated with fiction reading time when the model explicitly incorporated the long-tailedness of the RMET score.

**Table 2:** Bivariate correlations (*p*-values) between various activities including fiction/nonfiction reading and RMET/dispositional empathy (Study 1).

	Log_10_ Transformed Score						
	Fiction	Nonfiction	Sports	Videogame	*Manga*	Film	SNS^b^
RMET^a^	.11 (.052)	.02 (.661)	.08 (.140)	−.01 (.901)	.07 (.172)	.09 (.109)	.01 (.919)
Perspective Taking	.10 (.089)	.01 (.880)	.03 (.533)	.01 (.867)	.01 (.886)	.02 (.668)	−.08 (.121)
Personal Distress	−.03 (.634)	−.09 (.099)	−.01 (.918)	−.01 (.834)	−.04 (.515)	.04 (.500)	.11 (.042)
Empathic Concern	−.07 (.211)	−.02 (.743)	.07 (.202)	−.01 (.819)	−.12 (.032)	.03 (.553)	−.04 (.481)
Fantasy	.21 (< .001)	.03 (.305)	.03 (.635)	.03 (.642)	.15 (.006)	.19 (< .001)	.14 (.012)

*Note*. Significant correlations are indicated in bold font.

^a^RMET = “Reading the Mind in the Eyes Test” score.

^b^SNS = social networking services.

For exploratory purposes, additional analyses were conducted. First, our dataset allowed us to examine the replicability of another previous study [48], which reported that the association between fiction reading time and RMET score was not accounted for by individual differences in the tendency to be transported into stories (operationalized as the IRI FS score). Therefore, we also conducted a robust regression analysis in which the RMET score was the dependent variable and fiction reading time and FS were the independent variables. Conceptually replicating [48], the effect of fiction reading time was significant after controlling for the effect of FS: Bayesian 95% credible intervals were [.001, .23] and [−.03, .19] for fiction reading time and FS, respectively.

Second, we computed the correlation coefficients with the time spent on other activities: sports, videogames, *manga*, film, and social networking services (SNS). We included these items as fillers to obscure the purpose of the study. However, after data collection, we noticed that the SPaCEN framework [25] assumes that other forms of narrative, such as films, have effects similar to those of fiction (see [45] for a consistent finding). Therefore, we decided to also report the relevant correlations. As shown in Table 2, RMET and perspective taking did not correlate with any of these variables. Personal distress was positively correlated with the use of social networking services (SNS), *r*(335) = .11, *p* = .042. Empathic concern was negatively correlated with the time spent reading *manga*, *r*(336) = −.12, *p* = .032. More importantly, fantasy was positively correlated with time spent on *manga* (*r*(336) = .15, *p* = .006), film (*r*(336) = .19, *p* < .001), and SNS (*r*(335) = .14, *p* = .012). As trait fantasy is defined as a tendency to transpose oneself into the feelings of fictitious characters, it is not surprising that individuals high in fantasy spend time on *manga* and films.

Study 1 conceptually replicated previous findings [15, 48]: Reading fiction was positively associated with the RMET score (when the effect of an outlier was statistically controlled for) and IRI fantasy score. In addition, the association between fiction reading and RMET score was intact after controlling for the IRI fantasy score. However, contrary to Mumper and Gerrig’s meta-analysis [24], which found smaller but significant correlations between fiction reading time and other facets of IRI, as well as correlations between nonfiction reading time and empathic traits (including theory-of-mind), we found that these correlations were nonsignificant. Retrospectively, we conducted a sensitivity power analysis, which indicated that given the sample size of 338, the minimum effect size (*r*) that is associated with a power of 0.8 is .15. According to meta-analysis [24], there were only two effect sizes that exceeded this .15 threshold: the correlation between fiction reading and FS (.178) and the correlation between fiction reading and theory-of-mind (.211). Therefore, considering the sample size of this study, the entire pattern (i.e., fiction reading correlated only with FS and RMET) is consistent with the results of the meta-analysis [24]. Given the presence of associations between fiction reading and empathy/theory-of-mind, we proceeded to test the causal relationship between brief exposure to fiction and theory-of-mind ability, operationalized as the RMET score.

## Study 2

### Method

#### Design and participants

Study 2 was a conceptual replication of Kidd and Castano’s seminal experimental study [26]. However, we made the following modifications: First, we included repeated measures of RMET by dividing the 36 photographs into two sets of 18 photographs with equal difficulty (henceforth referred to as RMET-A and RMET-B), which allowed us to test whether brief exposure to short texts from fiction would improve RMET performance (see [21] for use of an 18-item version of RMET). Second, we dropped one condition to increase the sample size per condition (i.e., to achieve at least 100 participants in each condition). Project-wise, Kidd and Castano’s experiments included four conditions [26]: literary fiction, popular fiction, nonfiction, and no-reading (control) conditions. We dropped the popular fiction condition and retained the other three conditions. In this study, participants in the no-reading control condition engaged in a calculation task rather than doing nothing [26]. In sum, Study 2 employed a 3 (literary fiction vs. nonfiction vs. no reading) × 2 (before vs. after the reading/calculation task) factorial design involving repeated measures for the latter factor. However, we planned to analyze the difference between the after-task and before-task scores.

We did not conduct an a priori power analysis because we were unable to find the appropriate effect size for the difference score (see [34] for a within-participant study, which was not available when we conceived of Study 2; see also [27] for a different within-participant design study in which each participant read both fiction and nonfiction and engaged in the same RMET twice). Accordingly, we decided to include at least 100 participants in each condition. This sample size confers a power of 0.8, when comparing the two most relevant conditions (i.e., literary fiction vs. no reading) as long as the effect size (measured by Cohen’s *d*) exceeds .398 (i.e., small to medium-sized effect).

Participants were 304 undergraduate students at a large Japanese university: 154 women, 148 men, and 2 unreported; the mean age (*SD*) was 19.55 (1.10) years. The study was conducted from December 2017 to December 2018. They were assigned to three conditions in a semi-random manner (i.e., the number of participants per condition was balanced): *n*s = 102, 101, and 101 in the literary fiction, nonfiction, and control conditions, respectively. Participants were paid 1,000 Japanese yen for their participation.

#### Procedure and materials

Study 2 included the IRI, RMET-A, RMET-B, the exposure-to-fiction questionnaire (the same questionnaire used in Study 1), and a vocabulary test. Participants first completed the IRI questionnaire and then proceeded to REMT-A. Between RMET-A and RMET-B, participants in the fiction and nonfiction conditions read an excerpt from literary fiction or nonfiction for 30 minutes, while participants in the control condition engaged in a calculation task for 30 minutes.

The full set of RMET was divided into two sets (RMET-A and RMET-B), each consisting of 18 photographs. Based on the results of Study 1, we divided the 36 photographs such that the difficulty of the two sets would be nearly identical. The mean (*SD*) RMET-A and RMET-B scores in Study 1 were 12.77 (2.04) and 12.72 (2.15), *t*(337) = 0.34, *ns*. Nevertheless, as we will explain later, the performance of Study 2 participants was significantly better for RMET-A than for RMET-B.

After completing RMET-A, participants either read an excerpt from fiction or nonfiction (in the literary fiction and nonfiction conditions, respectively) or engaged in a calculation task for 30 minutes. We aimed to make our literary fiction materials comparable to those used in Kidd and Castano’s original study [26]. After consulting an expert in Japanese literature, we chose the following six books for literary fiction and nonfiction materials (three books for each condition). The literary fiction materials were Haruki Murakami’s *Tokyo Kitan-Shu* (Five Strange Tales from Tokyo; ISBN: 978-4-10-100156-2) (pp. 9–42 in the Japanese version;), Yoko Tawada’s *Hikon* (Flying Soul or Flying Spirits; ISBN: 978-4-06-290173-4) (pp. 3–35, no English translation available), and Mieko Kanai’s *Ai no Seikatsu* (Love Life; ISBN: 978-4-06-197578-1) (pp. 8–55, no English translation available). The backgrounds of these three authors can be found in English on Wikipedia (https://en.wikipedia.org/wiki/Haruki_Murakami, https://en.wikipedia.org/wiki/Yoko_Tawada, https://en.wikipedia.org/wiki/Mieko_Kanai). Participants in the literary fiction condition read excerpts from one of the three books. Each excerpt contained a narrative, which the SPaCEN framework assumes to be a key component for engaging social cognition [25].

The nonfiction materials were Shoji Ito’s *Jagaimo no Sekaishi* (The World History of the Potato; ISBN-13: 978–4121019301) (pp. 1–39, no English translation available), Ryoichi Honda’s *Iwashi wa Doko e Kietanoka* (Where Have the Sardines Gone?; ISBN-13: 978–4121019912) (pp. 3–39, no English translation available), and Toru Nishigaki’s *Big Data to Jinko-Chino* (Big Data and Artificial Intelligence; ISBN-13: 978–4121023841) (pp. 3–37, no English translation available). Participants in the nonfiction condition read excerpts from one of the three books. None of the three excerpts contained personal narrative.

In both the literary fiction and nonfiction conditions, participants were explicitly asked to read the same materials again if they finished reading the excerpt within 30 minutes. In the control condition, participants solved a set of difficult division calculations (e.g., 36873489600 ÷ 13275, 625976832 ÷ 2001) for 30 minutes. Because the experimenters monitored whether participants were actually reading the excerpts or solving the calculation problems, we did not include manipulation check questionnaires.

After reading the excerpts or solving calculation problems, participants completed the RMET-B, filled out the questionnaire regarding their exposure to fiction (the same questionnaire used in Study 1), and took a vocabulary test. We compiled practice questions from three textbooks for *Goi Dokkairyoku Kentei* (the Test of [Japanese] Vocabulary and Reading Comprehension Skills). This test was included to evaluate the validity of our fiction reading measure because it was different from the measure used in Western studies (i.e., ART). It is expected that fiction reading time is positively correlated with vocabulary size.

### Ethics statement

This study was approved by the Ethics Committee on Research Involving Human Subjects of the Graduate School of Humanities, Kobe University, Japan. Written informed consent was obtained from all participants.

### Results and discussion

#### Hypothesis testing

Before testing whether the RMET score improved in the literary fiction condition, we first confirmed that the REMT-A score (i.e., the RMET score before engaging in the reading/calculation task) did not significantly differ across the three conditions: 13.15 (*SD* = 2.07), 13.53 (1.89), and 13.20 (2.16) in the control, fiction, and nonfiction conditions, respectively, *F*(2, 301) = 1.05, *p* = 0.353, η^2^ = .007.

**Fig 2:**
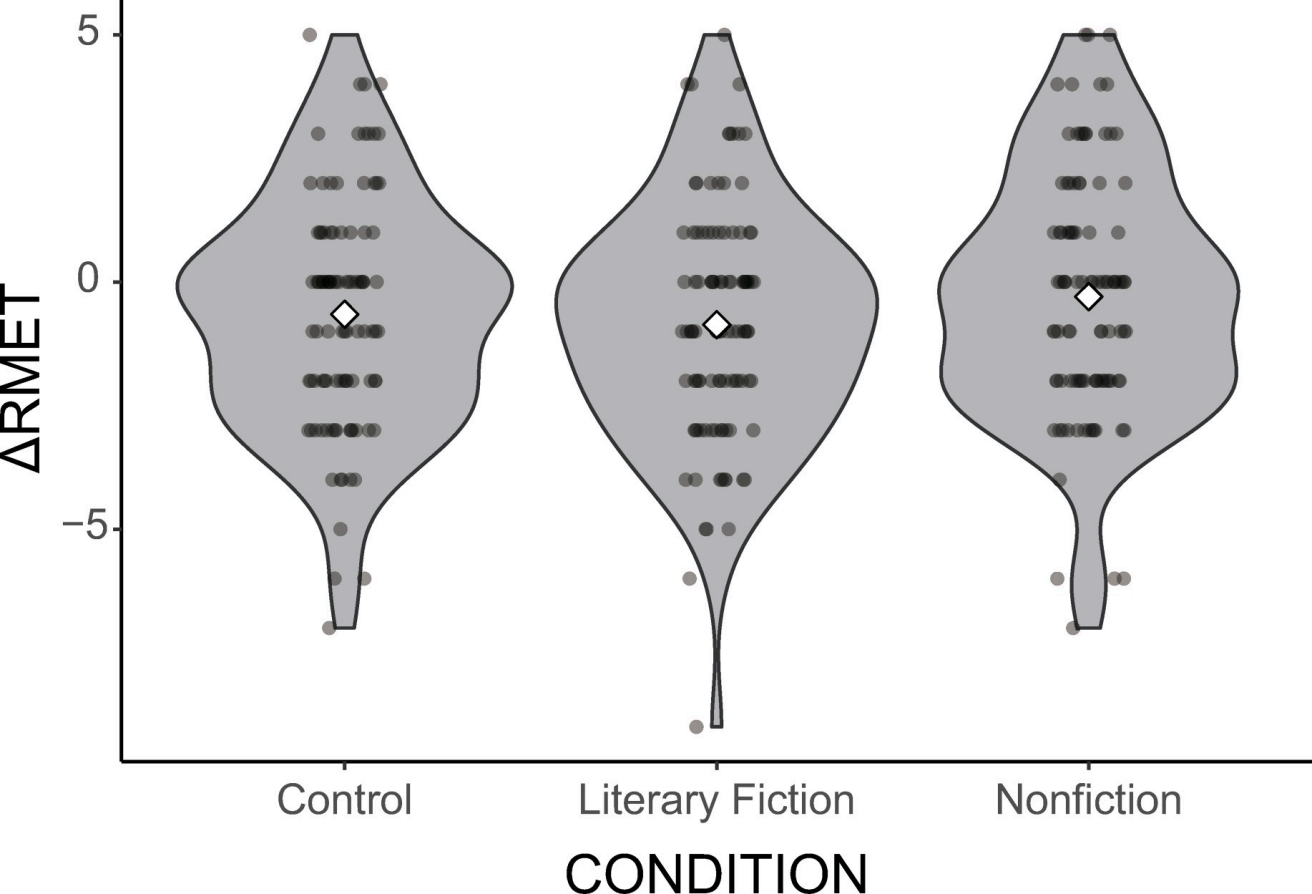
Violin plots of the ΔRMET distribution as a function of the experimental condition (Control, literary fiction, and nonfiction).

We then computed the difference between the pre- and post-reading/calculation task RMET scores (ΔRMET) by subtracting the RMET-A score from the RMET-B score. The distribution of ΔRMET as a function of the experimental manipulation is shown in Fig 2. A one-way analysis of variance (ANOVA) revealed a nonsignificant difference in ΔRMET: mean ΔRMET (*SD*) was −0.65 (2.34), −0.86 (2.40), and −0.30 (2.49) in the control, literary fiction, and nonfiction conditions, respectively, *F*(2, 301) = 1.43, *p* = .242, η^2^ = .009. Therefore, reading fiction for 30 min did not improve RMET performance compared to reading nonfiction and engaging in a calculation task. It is worth mentioning that the mean ΔRMET was not only negative but also lowest in the literary fiction condition, which was opposite to the hypothesized pattern (i.e., exposure to literary fiction, but not exposure to nonfiction or engaging in a calculation task, would increase the RMET score).

For exploratory purposes, we also conducted an ANOVA including gender as the independent variable because a previous meta-analysis revealed female superiority on RMET [49]. This 3 (condition) × 2 (gender) ANOVA with ΔRMET as the dependent variable again revealed a nonsignificant effect of condition, *F*(2, 296) = 1.47, *p* = .232, η_p_^2^ = .010. Although the effect of gender was not significant, *F*(1, 296) = 0.001, *p* = .977, η_p_^2^ < .001, we found an unexpected condition × gender interaction effect, *F*(2, 296) = 5.12, *p* = .004, η_p_^2^ = .036. As shown in S1 Table and S1 Fig in the S1 File, pairwise comparisons of six mean scores (three conditions × two gender categories) revealed only one significant difference: men’s ΔRMET was higher in the nonfiction condition (0.28, *SD* = 2.56) than in the literary fiction condition (−1.44, *SD* = 2.22). This pattern was contradictory to the original prediction: Reading literary fiction had a rather detrimental effect on men’s RMET performance as compared with reading nonfiction (cf. women’s ΔRMET was higher in the literary fiction condition than in the nonfiction condition, although it was not significant). This unexpected pattern must be interpreted with great caution because interaction effects without a priori hypotheses are associated with particularly low replicability [50]. Therefore, its replicability is yet to be confirmed.

#### Decrease in RMET

Unexpectedly, the mean ΔRMET scores were negative in all conditions (see [34] for a positive delta score when the same set of RMET was administered twice). A series of one-sample *t*-tests indicated that ΔRMET was significantly lower than zero in the control and literary fiction conditions: *t*(100) = −2.80, *p* = .006 and −0.86 (2.40), *t*(101) = −3.62, *p* < .001 in the control and literary fiction conditions, respectively; ΔRMET was also negative but not significantly different from zero in the nonfiction condition: *t*(100) = −1.20, *p* = .234. Given this unexpected decrease in RMET score, we tested the main hypothesis (i.e., exposure to literary fiction increases the RMET score) using the RMET-B score instead of ΔRMET. However, the RMET-B score did not significantly vary across the three conditions: 12.50 (*SD* = 2.19), 12.67 (2.02), and 12.91 (2.18) in the control, fiction, and nonfiction conditions, respectively, *F*(2, 301) = 0.93, *p* = 0.398, η^2^ = .006.

We speculated that this unexpected decrease in the RMET score could be due to the different difficulties in the two sets of RMET scores. To test this possibility, we recruited an additional 100 participants from the same undergraduate population: 52 women, 47 men, and 1 unreported; mean age (*SD*) = 19.31 (1.13) years. They completed two sets of RMET without any task between them. This new data, collected in 2019, showed a significant decrease in the RMET score even though there was no reading or calculation task between RMET-A and RMET-B: mean ΔRMET (*SD*) was −0.52 (2.20), *t*(99) = −2.36, *p* = .020.

When the new data were combined with the original Study 2 data, there was no significant effect of the four conditions (i.e., original “calculation” control, new “no task” control, literary fiction, and nonfiction conditions) on ΔRMET: *F*(3, 400) = 1.02, *p* = .382, η^2^ = .008. This nonsignificant result confirmed the original interpretation that reading fiction had no effect on RMET performance—reading fiction, even when compared with the no-task control, did not improve RMET performance.

#### Replication of Study 1

As an auxiliary analysis, we examined whether the results obtained in Study 1 could be replicated with Study 2 data. The dataset used in this section included an additional 100 participants in the no-task condition. Fiction reading time was operationalized as in Study 1 (log10 transformed). Fiction reading time was not correlated with any of the IRI subfactor scores. Even FS was not significantly correlated with fiction reading time, *r*(378) = .09, *p* = .094. We suspect that this attenuated correlation may be due to the experimental manipulation. When filling out the fiction reading questionnaire, some participants in the literary fiction condition might have thought that only fiction similar to the experimental materials that they had just read would be qualified as “fiction.” Although this is mere speculation, it should be noted that once the literary fiction condition was removed, the correlation between fiction reading time and FS became significant, *r*(286) = .15, *p* = .037.

Fiction reading time was not significantly correlated with the three variants of the RMET score: *r*(378) = .003, .006, and .006 for the RMET-A, RMET-B, and total RMET (i.e., RMET-A + RMET-B) scores, respectively (all *p*s > .40). As already noted, splitting the full RMET into two subsets had an unexpected effect on subset scores (i.e., RMET-A appeared to be easier than when it was embedded in the original full RMET). Therefore, the lack of a significant correlation between fiction reading time and the RMET score may be attributable to this procedural difference. Nonetheless, some researchers have cast doubt on the internal consistency and unidimensionality of the RMET (see [43, 51–53] for examples). Therefore, other theory-of-mind tests may be needed to adequately assess the short-term effects of fiction reading on theory-of-mind ability.

### Validity of fiction reading time

Although we operationalized fiction exposure differently from previous studies [15, 24], there is circumstantial evidence for the validity of our measure of fiction reading. We included a vocabulary test in Study 2 and found that fiction reading time was significantly correlated with the vocabulary test score, *r*(378) = .16, *p* = .002. This result suggests that individuals who spend a more time reading fiction have a larger vocabulary.

## General discussion

This study was the first attempt to conceptually replicate the relationship between fiction reading and dispositional empathy/theory-of-mind ability in an Asian country. Study 1 confirmed the previously reported *correlation* between fiction reading and empathy-related constructs [15, 24] among a Japanese undergraduate sample. In particular, we found a significant correlation between self-reported fiction reading time and IRI fantasy score. Although the correlation between self-reported fiction reading time and RMET failed to reach the conventional level of statistical significance, this was due to the presence of an outlier—the correlation became significant at the conventional .05-level when we used an estimation method that was robust against the presence of outliers. Despite the successful replication of the correlation between fiction reading and theory-of-mind, Study 2 failed to replicate the *causal relationship* reported by Kidd and Castano’s experimental study [26]: In the present study, a brief exposure to literary fiction did not enhance RMET performance.

A relatively robust pattern not only in this study but also in other studies summarized in Mumper and Gerrig’s meta-analytic review [24] is the correlation between fiction reading and IRI fantasy score. Although this correlation may appear rather self-evident given the definition of trait fantasy (i.e., a tendency to transpose oneself into the feelings and actions of fictitious characters), some studies suggest that it is more important than it may first appear. In a recent Japanese study [54], which used the same Japanese version of the IRI [40] as the present study, revealed that the fantasy score, but not the other three factors of IRI, was associated with higher performance on the empathic accuracy task [55], which is a more ecologically valid assessment of one’s mind-reading ability (i.e., participants are asked to infer thoughts and feelings of others in filmed conversations, and their inferences are scored based on the agreement with the filmed targets’ self-reported thoughts and feelings). Moreover, another intriguing experiment revealed that participants who were transported into an affective story tended to behave more prosocially toward an unrelated target [56]. Accordingly, the correlation between fiction reading and fantasy seems to have some implications for *everyday* mind-reading and empathy-based prosociality.

### Failure to replicate the causal relationship

Study 2 failed to conceptually replicate Kidd and Castano’s [26] finding. In Study 2, 30-min of exposure to fiction did not enhance RMET score. This result is in line with recent studies reporting replication failures [31–34]. In other words, there is a possibility that fiction reading has no causal effect on theory-of-mind ability. However, it is also possible that Study 2 failed to detect the hypothesized effect due to some procedural inadequacy.

First, unlike the original study [26], we split the 36 photographs of the RMET into two subsets to strictly test the improvement of the RMET score. However, previous studies have found the internal consistency of the RMET to be rather low, thus questioning the unidimensionality of the measure (see [43, 51–53] for examples). In fact, the correlation between RMET-A and RMET-B was low: *r*(402) = .30. Although significant at the .001-level, it appears to be too small to be regarded as the correlation between two measures assessing the same construct, the theory-of-mind ability in this case. Accordingly, Kidd and Castano’s causal relationship could have been replicated if we had used a more valid measure of theory-of-mind ability.

Second, there seem to be unidentified boundary conditions for Kidd and Castano’s original results [26]. For example, van Kuijk et al. [29] successfully replicated the original results when they used the same exclusion criterion used as in [26]. However, they failed to replicate it when they used a slightly different exclusion criterion (see also the exchange between Kidd and Castano [57] and Panero et al. [58] for the sensitivity of the result to exclusion criteria). Furthermore, one study [59] reported that fiction reading manipulation had a hypothesized effect on RMET when and only when other individual difference variables, such as openness or exposure to literature, were statistically controlled for. Therefore, it is possible that the causal effect of fiction reading on theory-of-mind ability is associated with some unspecified boundary conditions.

Third, we acknowledge that there are other subtle procedural differences. For example, we forced all participants to read the assigned excerpt for 30 minutes, while participants in the original study [26] proceeded to the RMET immediately after they finished reading the assigned excerpt. Such subtle procedural differences may be responsible for replication failure.

### Limitations and future directions

As we already noted, Studies 1 and 2 were not exact replications of the previous studies. This is a limitation of the present study. Nevertheless, from a different perspective, this limitation can also be a strength. For example, we did not use ART, which assesses respondents’ familiarity with fiction authors [16], because there was no readily available, validated Japanese version. Instead of assessing their familiarity with fiction authors, we directly asked participants to report the amount of time they usually spent reading fiction. This measure is conceptually equivalent to what the ART assesses but relies on a completely different method of assessment. Nevertheless, Study 1 succeeded in conceptually replicating the correlational results of Mar et al. [15]. We anticipate that there are many regions in which a local language version of ART is not available. The results of Study 1 encourage researchers in such regions to conduct conceptual replication studies using a seemingly crude measure of fiction exposure. However, it is also necessary to develop a local language version of ART following the procedure used in the original ART study [16] and subsequent extension studies [45, 60].

Unlike Kidd and Castano’s original study [26], Study 2 did not include online community samples. We decided to conduct Study 2 in small group sessions to ensure experimental manipulation (e.g., the in-person format of small group sessions allowed the experimenters to directly monitor whether participants were actually reading the assigned excerpts). However, this format forced us to rely exclusively on a student sample. That said, we recruited participants from a wide range of majors: humanities (19), liberal arts and education (53), law (30), economics (23), management (35), natural science including mathematics (30), engineering (40), agriculture (35), health science including medicine (25), and marine science (13). However, future replication studies should include Japanese (or Asian) community samples.

Another limitation is related to the validity of RMET. A recent study based on Item Response Theory (IRT) revealed that RMET may not be sufficiently sensitive to detect the effects of experimental manipulations among nonclinical samples [51]. Moreover, RMET performance is known to be positively associated with vocabulary (our measure of vocabulary was also significantly, but weakly, correlated with RMET score: *r*_402_ = .098, *p* = .049). This may be because RMET requires a large vocabulary to correctly identify mental state *descriptions*, but not mental states themselves. Consistent with previous studies (see [61] for an example), we found vocabulary to be positively correlated with fiction reading time. Therefore, even though the causal influence of exposure to fiction on RMET performance was replicable, there could be an alternative explanation: reading literary fiction temporarily enhances linguistic abilities but not theory-of-mind ability, and enhanced linguistic abilities allow respondents a more nuanced understanding of the mental state descriptions included in the RMET, which in turn promotes RMET performance. In future studies, other theory-of-mind tasks that do not depend on linguistic abilities need to be included. For example, Kidd and Castano [26] employed not only the RMET but also the Yoni task, which requires participants to figure out what an animated character thinks or wants. It would be interesting to conduct a conceptual replication of Kidd and Castano’s experiment using the Yoni task in Asian countries.

Study 2 revealed an unexpected interaction effect suggesting that reading literary fiction had a more detrimental effect on men’s RMET performance than reading nonfiction, whereas reading literary fiction had no such effect on women’s RMET performance. The replicability of this interaction effect must be confirmed before accepting it not just because the replicability of unexpected interaction effects is generally low [50], but also because it is contradictory to the results of previous meta-analyses: Both the meta-analysis of the causal effect of fiction reading [36] and the meta-analysis of fiction reading × theory-of-mind correlation [24] reported nonsignificant moderation effect of gender.

## Conclusion

This study successfully replicated the positive association of fiction reading with trait fantasy and theory-of-mind. To the best of our knowledge, this is the first replication study of this correlation in an Asian country. However, Study 2 failed to replicate the presumed causal relationship (i.e., brief exposure to fiction enhances theory-of-mind abilities). As empathy and theory-of-mind abilities are considered to be important building blocks of human sociality (or prosociality, to be more specific), and increasing time spent on fiction reading may provide a relatively low-cost intervention, it is important to deepen our understanding of the relationship between fiction reading and empathy/theory-of-mind in both Western and non-Western countries.

## Supporting information

S1 File (PDF)
