# Supplementary material for: Is reading fiction associated with a higher mind-reading ability? Two conceptual replication studies in Japan
Source: PLoS One. 2023 Jun 22;18(6):e0287542. doi: 10.1371/journal.pone.0287542 (PMC10286975; doi:10.1371/journal.pone.0287542)
Supplement: S1 File — (PDF) [file pone.0287542.s001.pdf]

<Supporting Information>
Is Reading Fiction Associated with a Higher Mind-Reading Ability? Two Conceptual Replication Studies in Japan

Study 1: AQ Score

Although we expected that the AQ score would be negatively correlated with the RMET (Reading the Mind in the Eyes test) score, the AQ score was not correlated with the RMET score: $r(336) = -.07$, $p = .171$). However, the AQ score was highly significantly correlated with three of the four facets of the IRI (interpersonal reactivity index): $r(336)$ was −.25, .27, and −.21 for perspective taking, personal distress, and empathic concern, respectively (all ps < .001). The correlation between AQ score and IRI fantasy was marginal: $r(336) = -.10$, $p = .064$. The correlational pattern between AQ and IRI was consistent with a previous study by Sindermann et al. (2019).

The AQ scores were not correlated with either fiction reading, $r(315) = .09$, $p = .129$, or nonfiction reading, $r(314) = .05$, $p = .341$. Moreover, it was not correlated with time spent on either videogame ($r(335) = .09$, $p = .0.93$), *manga* ($r(336) = .08$, $p = .137$) or films ($r(336) = -.08$, $p = .146$). However, individuals high in the AQ score tended to spend less time on sports ($r(336) = -.15$, $p = .005$) and SNS ($r(336) = -.14$, $p = .009$).

Reference

Sindermann, C., Cooper, A., & Montag, C. (2019). Empathy, autistic tendencies, and systemizing tendencies-relationships between standard self-report measures. *Frontiers in Psychiatry*, *10*, Article 307. https://doi.org/10.3389/fpsyt.2019.00307

Study 2: A 3 (Condition) × 2 (Gender) ANOVA

As mentioned in the main text, a 3 (condition) × 2 (gender) ANOVA for the Study 2 data indicated the significant interaction effect. Post-hoc comparisons indicated that the difference between the fiction and nonfiction conditions was significant (after being adjusted for multiple comparisons) only among men (Table S1 and Fig S1). Men's RMET score significantly decreased after reding fiction, as compared with nonfiction.

Table S1. Results of Tukey's HSD Tests.

Comparison			Difference	Adjusted *p*-value
Female Literary Fiction	vs.	Female Control	0.30	.988
Female Nonfiction	vs.	Female Control	-0.21	.998
Male Control	vs.	Female Control	-0.03	1.000
Male Literary Fiction	vs.	Female Control	-0.78	.588
Male Nonfiction	vs.	Female Control	0.93	.378
Female Nonfiction	vs.	Female Literary Fiction	-0.51	.883
Male Control	vs.	Female Literary Fiction	-0.33	.982
Male Literary Fiction	vs.	Female Literary Fiction	-1.09	.201
Male Nonfiction	vs.	Female Literary Fiction	0.63	.758
Male Control	vs.	Female Nonfiction	0.18	.999
Male Literary Fiction	vs.	Female Nonfiction	-0.57	.838
Male Nonfiction	vs.	Female Nonfiction	1.14	.158
Male Literary Fiction	vs.	Male Control	-0.76	.619
Male Nonfiction	vs.	Male Control	0.96	.339
Male Nonfiction	vs.	Male Literary Fiction	1.72	.006

Fig S1. Violin Plots of the ΔRMET Distribution as a Function of the Experimental Condition (Control, Literary Fiction, and Nonfiction Conditions) and Gender (Women and Men)

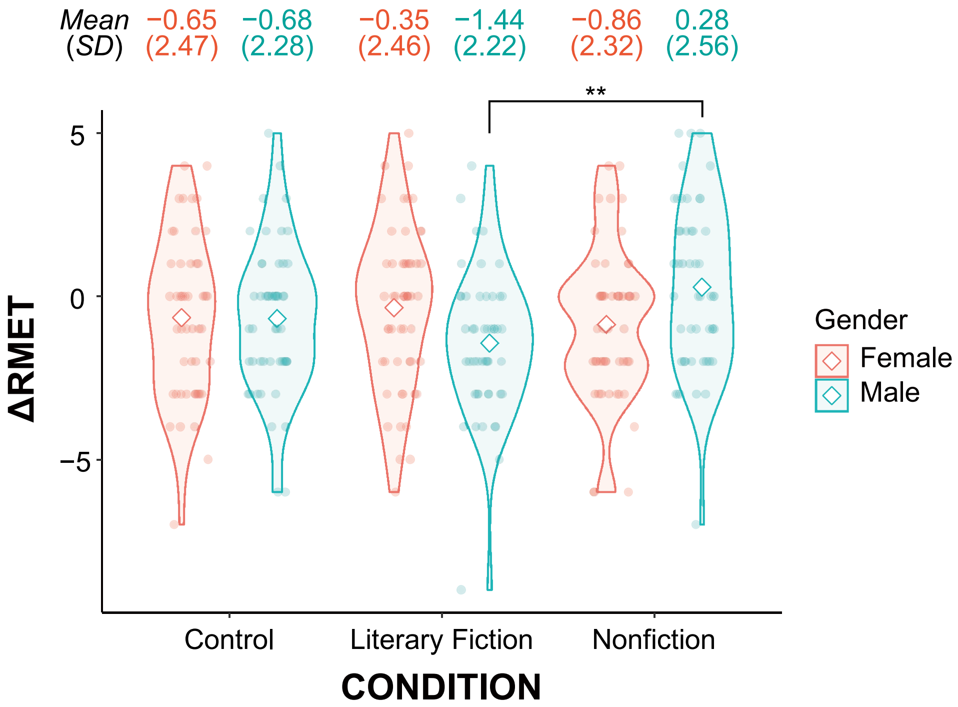

Notes. Main effect of condition: $F(2, 296) = 1.47$, $p = .232$, $\eta_p^2 = .010$. Main effect of gender: $F(1, 296) = 0.001$, $p = .977$, $\eta_p^2 < .001$. Interaction effect: $F(2, 296) = 5.12$, $p = .004$, $\eta_p^2 = .036$. Means (*SD*s) are shown in the top of the panel. Diamonds in violin plots indicate mean of the corresponding distributions.
